# High Expression of Ubiquitin-Specific Protease 39 and Its Roles in Prognosis in Patients with Hepatocellular Carcinoma

**DOI:** 10.1155/2021/6233175

**Published:** 2021-12-27

**Authors:** Yu Liao, Lin Li, Huabao Liu, Yi Song

**Affiliations:** Department of Hepatic Diseases, Chongqing Hospital of Traditional Chinese Medicine, Chongqing 400021, China

## Abstract

**Background:**

Ubiquitin-specific protease 39 is mainly involved in mRNA splicing and multiple kinds of tumors. Accumulating evidence has shown that USP39 participated in the proliferation and metastasis of hepatocellular carcinoma (HCC). The present study aimed to demonstrate the association between USP39 expression and clinical features and the diagnostic value in HCC based on the Cancer Genome Atlas (TCGA).

**Methods:**

A comprehensive analysis for expression of USP39 in HCC was conducted by using multiple databases. The mRNA level of USP39, clinical features, survival rate, and diagnostic value in HCC were analyzed using data from TCGA. The Gene Set Enrichment Analysis (GSEA) was conducted to analyze signaling pathways correlated with USP39 expression in HCC.

**Results:**

The mRNA level of USP39 was significantly elevated in HCC. The expression of USP39 showed significant correlation with T stage, pathologic stage, tumor status, age, and histologic grade. Logistic analysis demonstrated that high expression of USP39 was significantly associated with older age, tumor status, advanced pathologic stage, T stage, and higher histologic grade. Univariate Cox regression analysis showed that high expression of USP39 was significantly associated with advanced T stage, pathological stage, and tumor status. Multivariate Cox analysis confirmed the result that USP39 expression was an independent prognostic factor for overall survival (OS) in HCC. Results of Kaplan–Meier curves showed that high expression of USP39 had a significant association with poor OS, disease-free survival (DSS), and progress-free interval (PFI) in HCC. ROC analysis indicated that USP39 could be regarded as a promising marker for distinguishing HCC from nontumor.

**Conclusion:**

The increased USP39 might play roles in the progression, diagnosis, and prognosis of HCC.

## 1. Introduction

Hepatocellular carcinoma (HCC), mainly induced by a hepatitis virus infection, alcoholic consumption, or other liver diseases, is still among the most common malignancies worldwide. The 2018 global cancer statistics showed that the number of annual cases of HCC worldwide was 841,000, ranking sixth in the incidence spectrum of malignant tumors. Furthermore, the annual number of deaths from HCC was 781,000, ranking fourth among all the malignant tumors [1]. As for the treatment of tumors, the 5-year survival rate of 60%–70% can be obtained by surgical resection in patients with early-stage HCC, and the survival time can be prolonged by oral targeted drugs and immunotherapy [2]. In recent years, research on HCC mainly focused on regulating signaling pathways, tumor immunity, and targeted therapy in terms of tumor recurrence and metastasis. For instance, activation of Wnt signaling pathway, p53 pathway mutations, or Jak/STAT pathway has been reported to be related to the molecular features of HCC [3]. As for tumor immunity, activation of TGF-*β* signaling in HCC was reported to be significantly associated with immune cell exhaustion. The combination of TGF-*β* inhibitors and immune checkpoint inhibitors has emerged as a promising method in regulating tumor immune and microenvironment [4]. However, as far as the metastasis and recurrence of HCC are concerned, the effect of the above-mentioned treatment methods is still not satisfactory.

Deubiquitin enzymes (DUBs) are a large family of proteases, which hydrolyze ubiquitin molecules specifically from proteins linked to ubiquitin or precursor proteins by hydrolyzing the ester, peptide, or isopeptide bonding at the carboxyl end of ubiquitin. In humans, genes for deubiquitin include the cysteine proteases family and the metalloproteinase family. Among them, ubiquitin-specific proteases (USPs) belong to the family of cysteine proteases. USPs are a subfamily of the most important ubiquitin-modifying enzymes, which can reverse the regulation of protein degradation, the so-called deubiquitination. Recent studies have suggested that USPs are mainly involved in antivirus, immune regulation, and tumors. Viral infection can upregulate the expression of some USPs, such as USP5, USP7, and USP18, which affect the level of interferon [5–7]. USPs are involved not only in virus infection but also in tumors. For instance, USP10 promotes tumor metastasis through deubiquitination and stabilization of Smad4 in advanced HCC [8]. Through comprehensive bioinformatics analysis, Zhao et al. found that the transcription level of USP1 was elevated in HCC samples from the TCGA database. Moreover, overexpression of USP1 was correlated with higher grades, TP53 mutation, poor overall survival (OS), and immune infiltration [9]. As previously reported, the expression of USP39 was increased in HCC tumor tissues compared with that in adjacent nontumor tissues. Ni et al. reported that USP39 gene expression was significantly increased in HCC, and high expression of USP39 was correlated with poor prognosis [10]. The depletion of USP39 could inhibit the proliferation and metastasis of HCC cells [11]. Although USP39 is a member of the USPs family, it has no ubiquitin-specific peptidase activity. In recent years, it has been reported that USP39 was involved in tumor proliferation and metastasis through a variety of other mechanisms. These reports also suggested that USP39 might play vital roles in HCC. Therefore, exploring the roles of USP39 in HCC has become increasingly valuable.

Accordingly, in this study, we mainly enrolled public data from The Cancer Genome Atlas (TCGA) to evaluate the expression and clinical features of USP39 in HCC. Furthermore, we assessed the prognostic and diagnostic value of USP39 and explored the enriched signaling pathways in HCC. The above analyses preliminary elucidated the roles of USP39 in the diagnosis and prognosis of HCC, thus providing new ideas to improve understanding of USP39 in HCC.

## 2. Materials and Methods

### 2.1. Expression Level of USP39 in HCC

The gene expression of USP39 in different types of human cancers in TIMER (http://timer.comp-genomics.org/) [12] online database was analyzed. The *P* < 0.05 was considered to indicate a statistically significant result. The online cancer microarray database Oncomine (https://www.oncomine.org/) [13], an internet-based bioinformatics platform, was utilized to analyze the mRNA level of USP39 in HCC specimens and normal liver tissues. A fold change of 1.5, *P* < 0.05, and a gene rank in the top 10% were considered to indicate a statistically significant difference. The *P* value was calculated using Student's *t*-test. The Human Protein Atlas (HPA) (https://www.proteinatlas.org/) [14] is an open-access online database containing the pathology data for exploration of the human proteome. The level of USP39 protein in liver cancers and normal liver tissues was obtained from the HPA. The publicly open-access database TCGA (https://portal.gdc.cancer.gov/) was used to explore the expression of USP39 in HCC. The mRNA expression dataset with a total of 424 samples (Type: RNA-Seq FPKM) and the corresponding clinical information in TCGA were included. After excluding the incomplete samples, a total of 374 HCC samples were included for further analysis. In addition, Gene Expression Omnibus (GEO) (https://www.ncbi.nlm.nih.gov/geo/) [15], a public functional genomics data repository, was used to download gene expression datasets GSE45267 and GSE62232 about HCC to verify the mRNA expression of USP39.

### 2.2. Clinical Features from TCGA

The TCGA database was used to explore the correlation between mRNA expression of USP39 and clinical features, as well as the prognostic values of USP39 in HCC. Samples were divided into high and low groups according to the expression of USP39 above the median value and below the median value. Wilcoxon signed-rank test, chi-square test, and logistic regression were performed to analyze the association between clinical features including age, gender, TNM stage, histologic grade, pathologic stage, and residual tumor and USP39 mRNA expression in HCC. Univariate Cox analyses were performed to assess the effect of USP39 expression on predicting the potential prognostic factors on clinical outcome and survival. Furthermore, multivariate analysis was used to verify the prognostic value of USP39 expression. The hazard ratio (HR) with a 95% confidence interval (CI) was measured to assess the risk of individual factors. *P* value < 0.05 was considered statistically significant.

### 2.3. Survival and Diagnostic Value of USP39 in HCC

Samples were divided into high and low groups according to the median expression of USP39. Kaplan–Meier analysis was performed to compare the OS, disease-free survival (DSS), and progress-free interval (PFI) between the differentially expressed USP39 groups. The area under the received operating characteristic (ROC) curve was used to assess the diagnostic value of USP39. All statistical analyses were performed using *R* statistical software (version 3.6.3). *P* value < 0.05 was considered statistically significant.

### 2.4. Gene Set Enrichment Analysis (GSEA)

The online database LinkedOmics (http://www.linkedomics.org/) [16] was utilized to analyze signaling pathways correlated with USP39 expression in HCC. The gene set used in the present study was “c2.cp.v7.2.symbols.gmt”. Gene set permutation = 1000 times, *P* < 0.05, FDR <0.25, and normalized enrichment score (NES) > 1 were considered to indicate a statistically significant difference.

## 3. Results

### 3.1. Expression Level of USP39 in HCC

**Figure 1:**
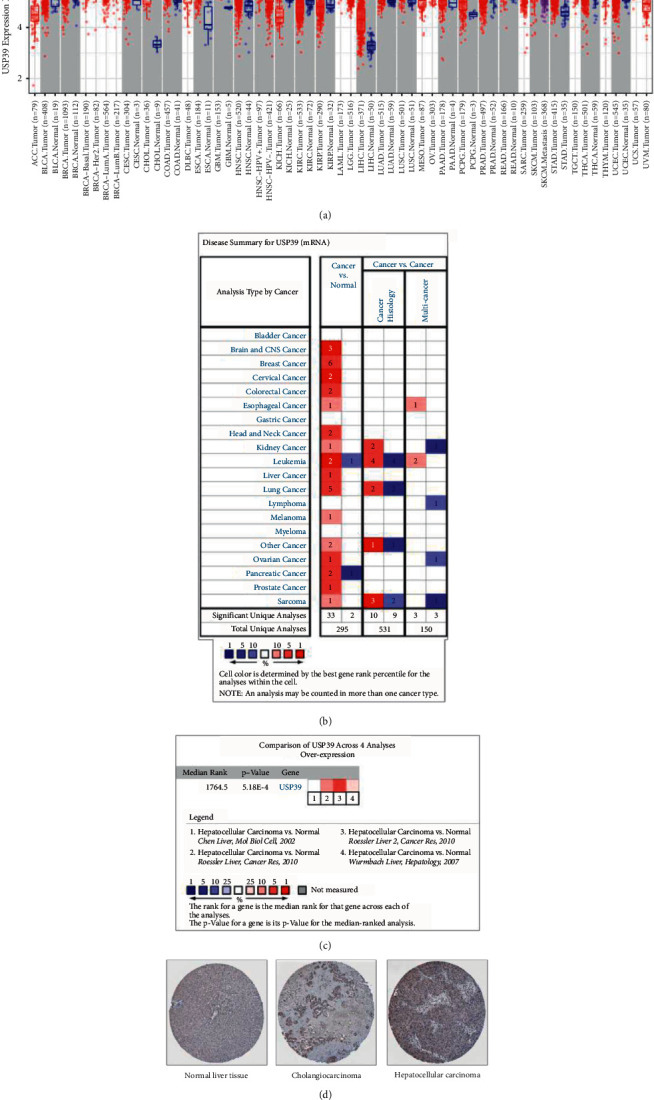
The expression level of the USP39 gene in different tumors in multiple databases. (a) The expression level of the USP39 gene in various human cancers from the TIMER database. The red columns represent tumors. The blue columns represent the corresponding normal tissues. (b) The expression level of the USP39 gene in various human cancers from the Oncomine database. The shade of the color represents the best gene rank percentile for the analyses. (c) The USP39 gene was significantly overexpressed in HCC from the Oncomine database. (d) The level of USP39 protein in cholangiocarcinoma, hepatocellular carcinoma, and normal liver tissue from the HPA (Antibody HPA077350, 10×).

Exploration of the level of USP39 in various human cancers was carried out in TIMER and Oncomine. Both Oncomine and TIMER databases showed an increase in the USP39 gene expression in HCC (Figure 1(a) and Figure 1(b)). Pooled analysis in the Oncomine database showed that USP39 was significantly overexpressed in HCC (*P* = 5.18*e* − 4, Figure 1(c)). Meanwhile, the level of USP39 protein in liver cancers was increased compared with that in normal liver tissue from the HPA (Figure 1(d)).

**Figure 2:**
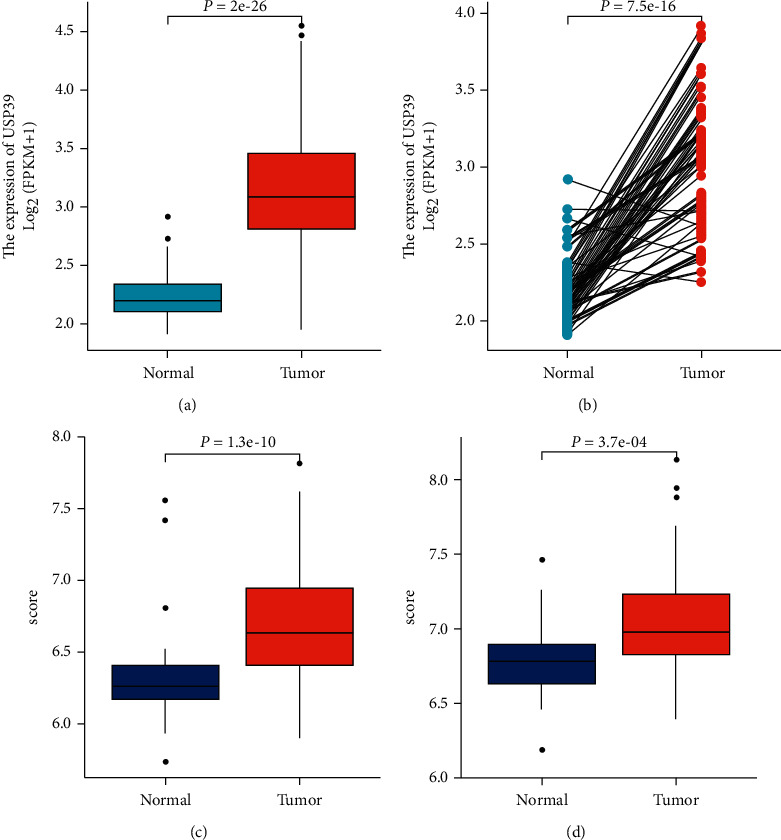
The expression level of USP39 in HCC from TCGA and GEO databases. (a) USP39 mRNA expression in HCC tissues was significantly elevated (*P* = 2*e* − 26). (b) USP39 mRNA expression in paired tissues (*P* = 7.5*e* − 16). (c) USP39 mRNA expression in HCC was significantly elevated from GSE45267 (*P* = 1.3*e* − 10). (d) USP39 mRNA expression in HCC was significantly elevated from GSE62232 (*P* = 3.7*e* − 4).

To potentiate the reliability of the results, we further analyzed the mRNA level of USP39 in HCC from the TCGA database. Based on the results, the expression level of the USP39 gene was significantly elevated in HCC samples compared with normal liver tissues (*P* = 2*e* − 26, Figure 2(a)). The results were also confirmed in HCC samples and paired normal liver tissues (*P* = 7.5*e* − 16, Figure 2(b)). In addition, the expression level of the USP39 gene in HCC was validated using two gene datasets from GEO (*P* = 1.3*e* − 10 and *P* = 3.7*e* − 04, Figures 2(c) and 2(d)).

### 3.2. Correlation between USP39 Expression and Clinical Features

**Figure 3:**
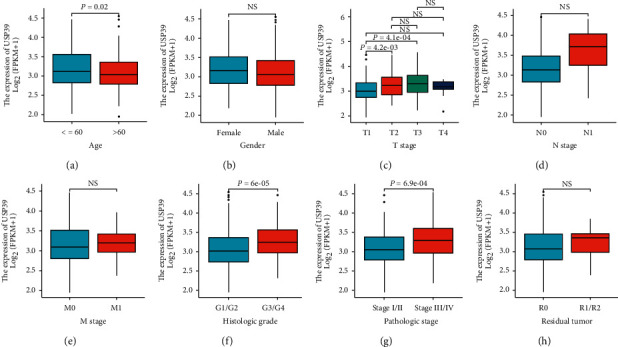
Correlation analysis of USP39 expression and clinical features in HCC. The increased USP39 expression level was correlated with age, histologic grade, and pathologic stage. (a) Age. (b) Gender. (c) T stage. (d) N stage. (e) M stage. (f) Histologic grade. (g) Pathologic stage. (h) Residual tumor. NS: no significance.

We also addressed the question of what relationship was between USP39 expression and clinical features using the TCGA database. Results in Figure 3 showed that increased USP39 expression level was correlated with age (*P* = 0.02), histologic grade (*P* = 6*e* − 05), and pathologic stage (*P* = 6.9*e* − 04).

Then, the HCC samples were divided into high and low groups based on the medium USP39 mRNA expression. The clinical features, including age, gender, stage (T, N, and M), pathologic stage, tumor status, residual tumor, and histologic grade, with differential expression of USP39 were summarized in Table 1. T stage (*P* = 0.006), pathologic stage (*P* = 0.009), tumor status (*P* = 0.006), age (*P* = 0.043), and histologic grade (*P* = 0.002) were significantly correlated with high USP39 mRNA expression level.

Results of logistic regression analysis demonstrated that USP39 expression level was associated with poor prognostic clinical features (Table 2). High expression of USP39 was significantly associated with tumor status (with tumor vs. tumor free: OR = 1.851, 95% CI = 1.212–2.840, *P* = 0.005), advanced pathologic stage (Stage III/IV vs. Stage I/II: OR = 2.047, 95%CI = 1.256–3.377, *P* = 0.004), advanced T stage (T3/T4 vs. T1/T2 : OR = 1.914, 95%CI = 1.188–3.118, *P* = 0.008), and higher histologic grade (G3/G4 vs. G1/G2 : OR = 2.148, 95%CI = 1.399–3.324, *P* <  0.001). Univariate analysis with Cox regression showed that high expression of USP39 (HR = 1.775, 95%CI = 1.250–2.519, *P* = 0.001), advanced *T* stage (HR = 2.540, 95%CI = 1.785–3.613, *P* < 0.001), advanced pathological stage (HR = 2.449, 95%CI = 1.689–3.549, *P* < 0.001), and tumor status (HR = 2.361, 95%CI = 1.620–3.441, *P* < 0.001) were significantly associated with poor OS (Table 3). Multivariate analysis confirmed that the expression of the USP39 gene was an independent prognostic factor for OS in HCC (HR = 1.539, 95%CI = 1.038–2.281, *P* = 0.032) (Table 3).

### 3.3. Roles of USP39 in Survival of HCC

**Figure 4:**
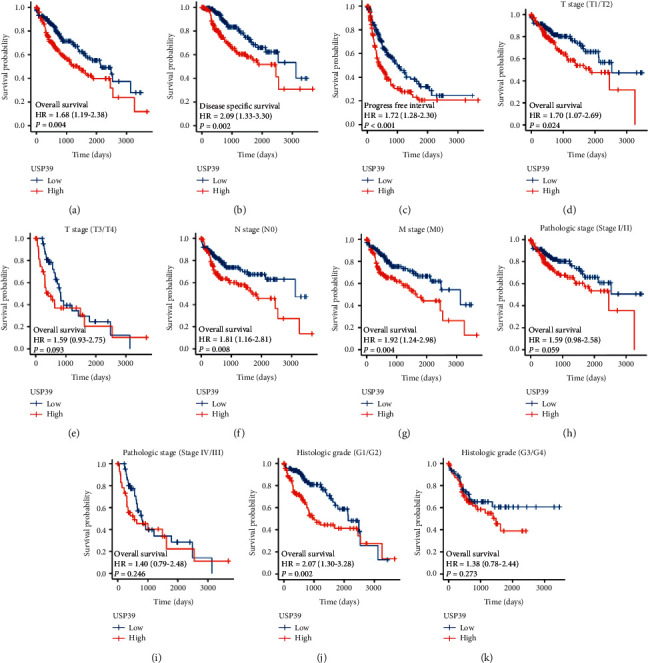
Kaplan–Meier curves for OS, DSS, and PFI in patients with HCC. High expression of USP39 was closely correlated with poor OS, DSS, and PFI in HCC. (a) Overall survival. (b) Disease-free survival. (c) Progress-free interval. High expression of USP39 was closely correlated with poor OS in T stage (T1/T2), N stage, M stage, and histologic grade. (d) T stage (T1/T2). (e) T stage (T3/T4). (f) N stage (N0). (g) M stage (M0). (h) Pathologic stage (stage I/II). (i) Pathologic stage (stage IV/III). (j) Histologic grade (G1/G2). (k) Histologic grade (G3/G4). HR: hazard ratio.

Subsequently, the roles of USP39 in HCC patients' survival were assessed (Figure 4). Results indicated that high expression of USP39 was correlated with poor OS, DSS, and PFI in HCC (*P* < 0.05). Furthermore, we assessed the OS prognostic values of USP39 in different subtypes of HCC, including T stage, N stage, M stage, pathological stage, histologic grade, residual tumor, tumor status, and vascular invasion, using Kaplan–Meier analysis. As shown in Figures 4(d)–4(k), elevated level of USP39 was associated with poor OS in T stage (T1/T2, *P* = 0.024), N stage (N0, *P* = 0.008), M stage (M0, *P* = 0.004), and histologic grade (G1/G2, *P* = 0.002). Taken together, these results indicated that the expression of USP39 could serve as a marker for predicting survival for different subtypes of HCC patients.

### 3.4. Diagnostic Value of USP39 in HCC

**Figure 5:**
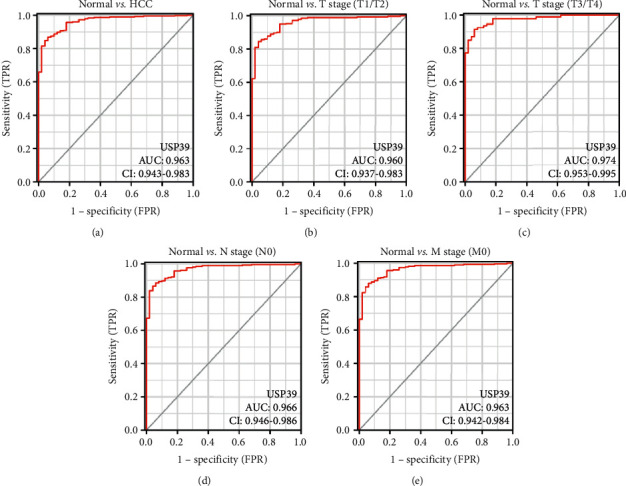
The expression of USP39 has a high diagnostic value in HCC. (a) ROC curve for USP39 in normal liver and HCC. (b) T stage (T1/T2). (c) T stage (T3/T4). (d) N stage (N0). (e) M stage (M0). AUC: area under the curve; CI: confidence interval; TPR: true positive rate; FPR: false positive rate.

ROC curve analysis was conducted to assess the distinguish ability of USP39 expression in HCC. The area under the curve (AUC) was 0.963, indicating a high diagnostic value of USP39 in HCC (Figure 5(a)). In addition, subgroup analyses showed similar diagnostic value of USP39 expression with AUC values of 0.960, 0.974, 0.966, and 0.963 for *T* stage (T1/T2), T stage (T3/T4), N stage (N0), and M stage (M0), respectively (Figures 5(b)–5(e)).

### 3.5. USP39-Related Signaling Pathways from GSEA

**Figure 6:**
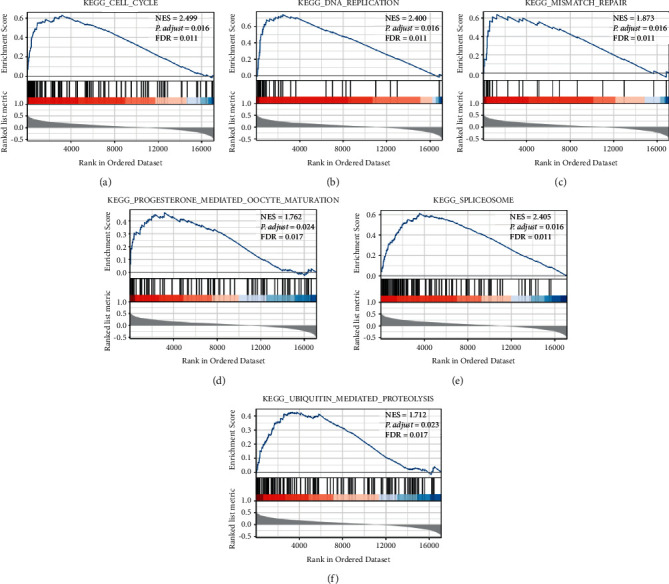
Enrichment plots by GSEA with USP39 expression phenotype in HCC. (a) Cell cycle. (b) DNA replication. (c) Mismatch repair. (d) Progesterone mediated oocyte maturation. (e) Spliceosome. (f) Ubiquitin-mediated proteolysis. NES: normalized enrichment score; FDR: false discovery rate.

To elucidate the related signaling pathways of USP39 in HCC, GSEA analysis was performed. The results demonstrated that signaling pathways such as cell cycle, DNA replication, mismatch repair, progesterone mediated oocyte maturation, spliceosome, and ubiquitin-mediated proteolysis, were significantly differentially enriched in the positively correlated with a phenotype of USP39 expression (Figure 6). Genes associated with the expression of USP39 are listed in Supplementary Table 1.

## 4. Discussion

HCC is one of the most common malignant liver tumors with high morbidity and mortality. Surgical resection or liver transplantation is the most effective treatment for early-stage HCC nowadays. However, due to the lack of effective diagnostic methods in the early stage and the lack of in-depth understanding of the pathogenesis of this disease, HCC patients were usually diagnosed at an advanced stage [17, 18]. Therefore, it is important to explore the key molecules in the occurrence and development of HCC and develop effective diagnostic and therapeutic markers. The ubiquitin-proteasome pathway is a multifunctional protein posttranslational modification involved in many cellular activities in the human body. The ubiquitin regulation of protein is a reversible process, which can be negatively regulated by some specific deubiquitin proteases [19]. USPs are one of the most important subfamilies of the deubiquitinase family. In recent years, studies have confirmed that the abnormal expression of USP39 was closely related to the occurrence and development of tumors [20, 21]. However, the prognosis and biological characteristics of USP39 in HCC remain to be a mystery. Therefore, we speculate that the role of USP39 in HCC needs to be further explored.

In this study, results from multiple databases showed that USP39 was differentially expressed in liver cancer tissues compared with normal tissues. Furthermore, an exploration of the association between the expression of USP39 and clinical features in HCC samples from TCGA was carried out. Results showed that high expression of USP39 was associated with T stage, pathologic stage, tumor status, age, and histologic grade. The expression of USP39 was not only elevated in many kinds of tumors but also associated with poor prognosis. A previous study showed that high expression of USP39 was associated with poor survival of patients with leukemia [22]. In ovarian carcinoma, a high level of USP39 mRNA and protein was associated with advanced tumor stage, poor OS, and progression-free survival [21, 23]. In pancreatic cancer, USP39 was correlated with TNM stage, depth of invasion, and poor survival [24]. A previous study reported that USP39 was significantly elevated in HCC as compared with nontumor tissues and was correlated with poor prognosis [10]. In the present study, logistic analysis demonstrated that high expression of USP39 was significantly associated with tumor status, advanced pathologic stage, T stage, and high histologic grade, which was basically consistent with those of the previous studies [10, 25]. Cox regression analysis confirmed the result that USP39 expression was an independent prognostic factor for OS in HCC. Prognostic analysis using Kaplan–Meier curves showed that high expression of USP39 had a significant association with poor OS, DSS, and PFI in HCC. ROC analysis indicated that USP39 could be regarded as a promising marker for distinguishing HCC from nontumor. Taken together, these results indicated that USP39 could serve as a marker for predicting survival for different subtypes of HCC patients.

The results of GSEA analysis showed that gene sets such as cell cycle, DNA replication, mismatch repair, progesterone mediated oocyte maturation, spliceosome, and ubiquitin-mediated proteolysis, were positively associated with USP39 expression phenotype. The depletion of USP39 has been found to cause activation of the p53 signaling pathway, an important regulatory mechanism in cell cycle and DNA replication, by stabilizing the p53 target p21 in multiple kinds of cancer cell lines [23, 26, 27]. Thus, USP39 could be regarded as an oncogene by promoting the progression and metastasis of tumors. Previous studies have shown that USP39 was mainly involved in pre-mRNA splicing as a component of the U4/U6.U5 tri-snRNP [28, 29]. Knockdown of USP39 could inhibit the proliferation and induce apoptosis of SMMC-7721 cells, as well as the growth of xenograft tumors in nude mice. A mechanism study suggested that knockdown of USP39 induced apoptosis by downregulating the pre-mRNA splicing of FoxM1 [30]. Thus, depletion of USP39 could inhibit the proliferation and metastasis of HCC cells. A further mechanism exploration indicated that USP39 could regulate the stability of ZEB1, a crucial inducer of epithelial-to-mesenchymal transition (EMT), by interacting with the E3 ligase TRIM26 directly, thus regulating the proliferation and metastasis of HCC [11]. Although it was reported that USP39 did not have ubiquitin-specific peptidase activity [31], it contained a ubiquitin-protease domain, which allowed it to inhibit the degradation of ZEB1 through deubiquitination activity and thus promote the progression of HCC. However, a few studies about other signaling pathways are related to USP39 in HCC. For this reason, further exploration of USP39 and its related signaling pathways in HCC is needed.

## 5. Conclusion

In the present study, we explored the expression level of USP39 and identified its roles in the prognosis of HCC, which might help broaden our understanding of the roles of USP39 in tumorigenesis and diagnosis of HCC. We mainly analyzed the transcription level changes of USP39, but not in the posttranscription level. Nevertheless, the above analyses might provide new insight into the prognosis and biological characteristics of USP39 in HCC.

## Figures and Tables

**Table 1:** Clinical features with differentially expressed USP39 in HCC.

Characteristic	*n*	Low expression of USP39	High expression of USP39	*P*
*T stage, n (%)*				0.006
T1	183	107 (58.2%)	76 (40.6%)	
T2	95	42 (22.8%)	53 (28.4%)	
T3	80	31 (16.8%)	49 (26.2%)	
T4	13	4 (2.2%)	9 (4.8%)	

*N stage, n (%)*				0.624
N0	254	122 (99.2%)	132 (97.8%)	
N1	4	1 (0.8%)	3 (2.2%)	

*M stage, n (%)*				0.623
M0	268	132 (99.2%)	136 (97.8%)	
M1	4	1 (0.8%)	3 (2.2%)	

*Pathologic stage, n (%)*				0.009
Stage I	173	100 (57.5%)	73 (41.5%)	
Stage II	87	41 (23.6%)	46 (26.1%)	
Stage III	85	31 (17.8%)	54 (30.7%)	
Stage IV	5	2 (1.1%)	3 (1.7%)	

*Tumor status, n (%)*				0.006
Tumor free	202	114 (64.4%)	88 (49.4%)	
With tumor	153	63 (35.6%)	90 (50.6%)	

*Gender, n (%)*				0.185
Female	121	54 (28.9%)	67 (35.8%)	
Male	253	133 (71.1%)	120 (64.2%)	

*Age, n (%)*				0.043
≤ 60	177	78 (41.9%)	99 (52.9%)	
>60	196	108 (58.1%)	88 (47.1%)	

Median (IQR)	123	64 (54, 70)	59 (51, 67)	0.005

*Residual tumor, n (%)*				0.083
R0	327	170 (96.6%)	157 (92.9%)	
R1	17	5 (2.8%)	12 (7.1%)	
R2	1	1 (0.6%)	0 (0.0%)	

*Histologic grade, n (%)*				0.002
G1	55	35 (18.9%)	20 (10.9%)	
G2	178	98 (53.0%)	80 (43.5%)	
G3	124	49 (26.5%)	75 (40.7%)	
G4	12	3 (1.6%)	9 (4.9%)	

*Vascular invasion, n (%)*				0.072
No	208	118 (70.2%)	90 (60.0%)	
Yes	110	50 (29.8%)	60 (40.0%)	

IQR: interquartile range.

**Table 2:** Logistic analysis of correlation between USP39 expression and clinical features.

Characteristics	Total (*n*)	Odds ratio (OR)	*P* value
T stage (T3/T4 vs. T1/T2)	371	1.914 (1.188–3.118)	0.008
N stage (N1 vs. N0)	258	2.773 (0.350–56.463)	0.380
M stage (M1 vs. M0)	272	2.912 (0.368–59.271)	0.357
Pathologic stage (Stage III/IV vs. Stage I/II)	350	2.047 (1.256–3.377)	0.004
Tumor status (with tumor vs. tumor free)	355	1.851 (1.212–2.840)	0.005
Residual tumor (R1/R2 vs. R0)	345	2.166 (0.820–6.348)	0.131
Histologic grade (G3/G4 vs. G1/G2)	369	2.148 (1.399–3.324)	<0.001
Vascular invasion (yes vs. no)	318	1.573 (0.990–2.511)	0.056

**Table 3:** Univariate and multivariate Cox regression analyses of correlation between USP39 expression and clinical features.

Characteristics	Total (*n*)	Univariate analysis		Multivariate analysis	
		Hazard ratio (95% CI)	*P* value	Hazard ratio (95% CI)	*P* value
T stage (T3/T4 vs. T1/T2)	367	2.540 (1.785–3.613)	<0.001	1.696 (0.231–12.438)	0.603
Pathologic stage (Stage IV/III vs. Stage I/II)	346	2.449 (1.689–3.549)	<0.001	1.197 (0.164–8.733)	0.859
Tumor status (with tumor vs. tumor free)	351	2.361 (1.620–3.441)	<0.001	1.794 (1.196–2.691)	0.005
Gender (male vs. female)	370	0.816 (0.573–1.163)	0.260		
Age (>60 vs. ≤ 60)	370	1.248 (0.880–1.768)	0.214		
Residual tumor (R1/R2 vs. R0)	341	1.571 (0.795–3.104)	0.194		
Histologic grade (G3/G4 vs. G1/G2)	365	1.120 (0.781–1.606)	0.539		
Vascular invasion (yes vs. no)	314	1.348 (0.890–2.042)	0.159		
USP39 (high vs. low)	370	1.775 (1.250–2.519)	0.001	1.539 (1.038–2.281)	0.032

CI: confidence interval.

## Data Availability

All data generated or analyzed during this study are included in this article.

## Supplementary Materials

Supplementary Materials Supplementary Table 1 shows the GSEA analysis of genes associated with the expression of USP39.
