# Supplementary material for: High Expression of Ubiquitin-Specific Protease 39 and Its Roles in Prognosis in Patients with Hepatocellular Carcinoma
Source: Evid Based Complement Alternat Med. 2021 Dec 27;2021:6233175. doi: 10.1155/2021/6233175 (PMC8723855; doi:10.1155/2021/6233175)
Supplement: Supplementary Materials — Supplementary Table 1 shows the GSEA analysis of genes associated with the expression of USP39. [file 6233175.f1.docx]

Supplementary table 1: GSEA analysis of genes associated with the expression of USP39.

| Description | ES | NES | *P*. adjust | FDR *Q* value | Core enrichment |
| --- | --- | --- | --- | --- | --- |
| KEGG_CELL_CYCLE | 0.632 | 2.499 | 0.016 | 0.011 | BUB1/CDK1/DBF4/BUB1B/CCNB2/PLK1/CDC25A/TTK/CDK2/MCM3/MCM4/YWHAQ/CCNB1/MCM6/MCM2/ANAPC7/SMC3/CCNA2/CDK4/CDC45/ANAPC1/MCM7/CCNE1/SKP2/CDC20/MAD2L1/HDAC2/CDC6/PTTG1/E2F3/RBL1/ESPL1/CDC25C/CDC7/CHEK1/CCNE2/TFDP1/E2F2/E2F1/MCM5/ABL1/RAD21/PKMYT1/ANAPC4/PCNA/ANAPC5/PRKDC/CHEK2/BUB3/CDC23/E2F4/CDC14A/E2F5/STAG1/SMC1B/CDK7/GSK3B/CDKN2C/ATR/YWHAZ/SMAD2/HDAC1/PTTG2/SMAD3/CDC25B/YWHAH/CDC27/TP53 |
| KEGG_SPLICEOSOME | 0.613 | 2.405 | 0.016 | 0.011 | HNRNPU/ACIN1/TCERG1/RBM17/RBMX/U2AF2/SMNDC1/HNRNPA1L2/NCBP2/TRA2B/HNRNPA3/SNRNP200/SNRPG/THOC1/WBP11/SF3A2/PRPF40A/SF3B4/SNRPB/SNRPD1/DDX42/DDX23/SF3B1/RBM25/HNRNPK/SNW1/DHX15/HNRNPC/EFTUD2/DDX46/SNRNP40/PRPF40B/PRPF38B/EIF4A3/SNRPA1/HNRNPA1/RBM8A/HNRNPM/SNRNP27/SNRPA/SNRPF/THOC2/HSPA1L/PRPF3/ISY1/LSM2/THOC3/PRPF4/SNRPE/PRPF38A/PHF5A/DHX16/CRNKL1/U2AF1/SNRPB2/TRA2A/PCBP1/DHX8/AQR/NCBP1/PPIL1 |
| KEGG_DNA_REPLICATION | 0.740 | 2.400 | 0.016 | 0.011 | MCM3/MCM4/MCM6/MCM2/PRIM2/POLA1/RPA1/LIG1/MCM7/POLD1/RFC4/DNA2/RFC3/POLD3/RFC5/PRIM1/POLE2/MCM5/FEN1/PCNA/POLA2/RNASEH2A/RNASEH2B/RFC2/POLE3 |
| KEGG_MISMATCH_REPAIR | 0.639 | 1.873 | 0.016 | 0.011 | MSH2/EXO1/RPA1/LIG1/POLD1/RFC4/RFC3/POLD3/RFC5/MSH6/PCNA |
| KEGG_PROGESTERONE_MEDIATED_OOCYTE_MATURATION | 0.465 | 1.762 | 0.024 | 0.017 | BUB1/CDK1/CCNB2/PLK1/CDC25A/CDK2/CCNB1/ANAPC7/CCNA2/ANAPC1/MAD2L1/CDC25C/PKMYT1/PIK3R2/MAPK13/ANAPC4/MAPK9/ANAPC5/ADCY6/CDC23/MAPK1/BRAF/HSP90AB1/KRAS/SPDYA/PRKX/CDC25B/CDC27/MAPK3/RPS6KA3 |
| KEGG_UBIQUITIN_MEDIATED_PROTEOLYSIS | 0.429 | 1.712 | 0.023 | 0.023 | UBE2N/UBA2/UBE2C/ANAPC7/SAE1/UBE2E1/ANAPC1/SKP2/CDC20/PIAS3/CUL2/VHL/BRCA1/FANCL/UBE2D1/SMURF2/CBL/ANAPC4/UBE2O/BTRC/ANAPC5/PIAS1/UBE2S/FBXW8/PIAS4/CDC23/UBE2Z/HUWE1/UBE2E3/UBE2Q2/TRIM32/UBE2Q1/AIRE/PPIL2/UBE3B/MAP3K1/UBE2K/CUL3/TRIM37/UBE2I/UBA3/TRIP12/UBR5/CDC27/UBOX5/BIRC6/RNF7/UBE2D2/ERCC8 |

*ES*: Enrichment score; *NES*: Normalized enrichment score.
